# Urban-rural disparities and change in postnatal care use from 2016 to 2019 in Ethiopia: Multivariate decomposition analysis

**DOI:** 10.1371/journal.pone.0299704

**Published:** 2024-09-03

**Authors:** Melash Belachew Asresie, Amit Arora

**Affiliations:** 1 Department of Reproductive Health and Population Studies, School of Public Health, College of Medicine and Health Sciences, Bahir Dar University, Bahir Dar, Ethiopia; 2 School of Health Sciences, Western Sydney University, Penrith, NSW, Australia; 3 Translational Health Research Institute, Western Sydney University, Campbelltown, NSW, Australia; 4 Health Equity Laboratory, Campbelltown, NSW, Australia; 5 Discipline of Child and Adolescent Health, The Children’s Hospital at Westmead Clinical School, Faculty of Medicine and Health, The University of Sydney, Westmead, NSW, Australia; 6 Oral Health Services, Sydney Local Health District and Sydney Dental Hospital, NSW Health, Surry Hills, NSW, Australia; Nazarbayev University School of Medicine, KAZAKHSTAN

## Abstract

**Background:**

Postnatal care (PNC) is essential for early identification and management of life-threatening obstetric complications. Despite efforts by the Ethiopian government to improve maternal and child health service use, PNC service has remained low, and disparity across geographic locations is a major public health problem. This study aimed to investigate the change and contributing factors in PNC service use across geographical locations (rural-urban) and over time (2016 to 2019) in Ethiopia.

**Methods:**

We analyzed data on women who gave birth from the 2016 and 2019 Ethiopian Demographic and Health Surveys. A total of 6,413 weighted samples (4,308 in 2016 and 2,105 in 2019) were included in the analysis. A multivariate decomposition analysis technique was used to determine the change and identify factors that contributed to the change across geographical locations and over time. Statistical significance was defined at a 95% confidence interval with a p-value of less than 0.05.

**Results:**

The prevalence of PNC use was higher among urban residents, and the urban-rural disparity reduced from 32.59% in 2016 to 19.08% in 2019. The difference in the composition of explanatory variables was the only statistically significant for the urban-rural disparity in PNC use in both surveys. Specifically, female household heads (4.51%), delivery at a health facility (83.45%), and birth order of two to three (5.53%) and four or more (-12.24%) in 2016 significantly contributed to the urban-rural gap. However, in 2019, middle wealth index (-14.66%), Muslim religion (3.84%), four or more antennal care contacts (18.29%), and delivery at a health facility (80.66%) significantly contributed to the urban-rural gap. PNC use increased from 16.61% in 2016 to 33.86% in 2019. About 60% of the explained change was due to the difference in the composition of explanatory variables. Particularly, urban residence (-5.79%), a rich wealth index (2.31%), Muslim (3.42%), and other (-2.76%) religions, having radio or television (1.49%), 1–3 (-1.13%), and 4 or more (11.09%) antenatal care contacts, and delivery at a health facility (47.98%) were statistically significant contributors to the observed change. The remaining 40% of the overall change was due to the difference in unknown behaviors (coefficient) of the population towards PNC.

**Conclusions:**

There was a significant change in PNC service use by residence location and over time in Ethiopia, with urban women in both surveys being more likely to use PNC service. The urban-rural disparity in PNC uptake was due to the difference in the composition of explanatory variables, whereas the change over time was due to the change in both the composition of explanatory variables and population behavior towards PNC. Increased antenatal care contacts and delivery at a health facility played a major role in explaining the gap in PNC services across residences and over time in Ethiopia, highlighting the importance of stepping up efforts to enhance their uptake in rural settings.

## Background

Despite significant improvements in maternal and child health in recent years, there remains an unacceptably high number of maternal and neonatal deaths globally. A pregnant woman or newborn baby dies every 11 seconds around the world, but this could be prevented by providing skilled care before, during, and after childbirth [1]. In 2017, an estimated 295,000 women died worldwide as a result of pregnancy and its consequences, with heterogeneity across the regions. Sub-Saharan Africa accounted for two-thirds (66%) of all maternal deaths worldwide [1]. An estimated 5 million children under the age of five died globally in 2020, with nearly half of those deaths (47%) occurring during the neonatal period [2]. Children born in Sub-Saharan Africa are 14 times more likely to die than children born in averagely high-income regions before reaching their fifth birthday in 2020 [2]. The majority of maternal and neonatal deaths occur within the first 48 hours of life [2, 3]. Approximately two-thirds of maternal deaths occur within the first two days of life due to preventable and treatable factors [3]. A third of neonatal deaths occur within the first 24 hours of life, and three-quarters occur within the first seven days [2].

Ethiopia is one of the sub-Saharan African countries where maternal and neonatal mortality remains high. Maternal mortality in Ethiopia has been declining in recent decades, but the rate of decline has been slow. This reduction has plateaued in the last five years, maintaining a constant rate of 412 deaths per 100,000 live births since 2016. Moreover, even though Ethiopia successfully achieved the Millennium Development Goal related to reducing under-five mortality ahead of time, the proportion of neonatal mortality contributed to under-five mortality has increased, and the rate of neonatal mortality has also been rising recently, from 28 deaths per 1000 live births in 2016 to 33 deaths in 2019 [4, 5].

Despite rising coverage of maternity and child health services in Ethiopia, unchanged maternal mortality and lately increased neonatal mortality rates may be linked to poor use and inequity in the uptake of maternal healthcare [6–9]. Postnatal care (PNC), which begins immediately after the baby is delivered and lasts six weeks or 42 days, is an essential maternity care component recommended by the World Health Organization (WHO) [10]. All women and newborn babies should have their health checked within two days of giving birth to safeguard both the mother and the child, treat any complications that arise during the delivery, and offer the mother critical information on how to care for herself and her child. However, despite the tremendous effort in the last two decades, the uptake of PNC service use is low. Evidence suggests that less than half of women worldwide receive a PNC visit within two days of childbirth [11] and, more specifically, only 13% of women in Sub-Saharan Africa receive PNC visits within two days of childbirth [12].

The Ethiopian government has designed various initiatives to enhance maternal health services, such as the Health Extension Programme and various strategies as well as expanding healthcare infrastructure, training skilled birth attendants, promoting community awareness, and providing maternal service free of charge to ensure access to maternal and child health services at the grassroots level [13, 14]. However, in Ethiopia, less than one in every six women in 2016 and one in every three women in 2019 received PNC services within two days following delivery [4, 5]. Although around three-fourths of women got antenatal care services and half of women delivered in health facilities [4], the low uptake of PNC makes it critical to study the underlying factors of PNC uptake. Moreover, an urban-rural disparity in PNC service use was observed in all Ethiopian Demographic and Health Survey (EDHS) reports [4, 5]. Furthermore, the PNC service use trend in Ethiopia was stagnant until 2016 but increased by more than 17% between 2016 and 2019 [4, 5]. However, the driving factors for the observed change over time and urban-rural disparity have not been thoroughly investigated. Investigating the factors that drive changes in PNC use is critical to achieving sustainable Development Goal Three (SDG3): reducing maternal deaths to 70 per 100,000 live births, ending preventable deaths of newborns, and reducing neonatal mortality to 12 per 1000 live births by 2030 [15]. Moreover, understanding urban-rural disparity and identifying factors for disparity are paramount to achieving health equity and quality healthcare services on global public health agendas, as well as achieving universal health coverage by 2030 [16, 17].

Decomposition analysis based on nationally representative data is paramount to understanding the changes, and disparity as well as identifying the driving factors for changes at the national level, which is critical for policymakers to drive the change in PNC uptake and minimize disparity [18]. Therefore, based on the 2016 and 2019 EDHS, this study aimed to investigate the changes and contributing factors in PNC service use across residences and over time in Ethiopia.

## Methods

### Data source

We used the 2016 and 2019 EDHS data, which is administrated across nine regions and two cities. Ethiopia is located in the eastern part of the African continent and has the second-largest population in the African continent and the 12^th^-largest population in the world [4, 5].

### Study design and sampling technique

The two EDHSs were national, regional, and residential representative cross-sectional community-based surveys. The EDHS used a two-stage stratified cluster sampling technique. The Ethiopia Population and Housing Census (PHC), conducted in 2007, served as the sampling frame for each EDHS. The census frame is a comprehensive list of the 84,915 enumeration areas (EAs) established for the 2007 PHC. It includes information about the EA’s location, types of residence, and the expected number of residential households in each region. Each region was initially stratified into urban and rural areas, yielding 21 sampling strata. Enumeration areas (EAs) were chosen in the first stage with a probability proportional to EA size based on Ethiopia’s 2007 PHC at each survey. Then, in all of the selected EAs, household listing procedures were carried out. In the second stage of selection, households in the cluster were selected using an equal-probability systematic selection method. At each survey, women who were either permanent residents of the selected households or visitors who slept in the household the night before the survey were asked about PNC service use during their most recent live births [4, 5].

### Study population and sample size

The study participants for this study were women with the most recent births in the two years preceding the survey. A total of 6,413 weighted samples were included in the analysis: 4,308 from the 2016 EDHS (rural = 3,788, urban = 520); and 2,105 from the 2019 EDHS (rural = 1,552; urban = 553) [4, 5].

### Measurements

#### Outcome variables

This study’s outcome variable was PNC service use within the first two days after childbirth. It was defined as getting a postnatal checkup or visit during the first two days of childbirth. In the survey, women were asked, "After you gave birth, did you get any check-ups on your health?" If the answer was yes, how many days or weeks after the delivery did the first checkup take place? If the answer was within two days, we coded it as “Yes” and otherwise “No” to determine if the mother had attended any PNC visits or check-ups within the first two days after delivery. Then we coded "1" for the “Yes” response and "0" for the “No” response [4, 5].

#### Grouping variable

The survey year (2016 and 2019) and residence (urban and rural) were used to categorize study participants.

#### Explanatory variables

Based on their availability in two EDHS datasets and the literature [19, 20], the following variables were chosen as exploratory variables: Women’s ages (15–24, 25–34, and 35–49 years) at the time of the interview, educational status (no education, primary, and secondary or above), religion (Christian, Muslim, and other (others and traditional), marital status (in union and not in a union), sex of the household head (male and female), number of under-five children in the household (zero, one, and two or more), sex of the index child (male and female), birth order (1, 2–3, and 4 or more), place of birth (home, and health facility), antenatal care (ANC) contacts (no, 1–3, and 4 or more), having radio/television in the household (no and yes), and household wealth index (poor, middle and rich). The poor wealth index category was created by merging the poorest and poorer and the rich category by merging the richest and richer since some categories are too small for statistical analysis.

#### Data management and analysis

Using the "append" command in STATA software version 15.1, the 2019 EDHS data was appended to the 2016 EDHS data. To adjust for disproportionate sampling and non-response, normalized weight was used. We also used the "svyset" command to handle the effect of the complex sample survey in EDHS. After joining the two datasets, variables were recoded to create new categories. A multivariable decomposition non-linear model of analysis was performed to understand change across time and residence, as well as to identify factors that contributed to the change and disparity across time and residences. The model was used to estimate the difference between the two groups into components based on the observed characteristics between the groups and the predicted effects of the characteristics [18]. This provided detailed information about the source of the difference between the two groups due to changes in the composition of explanatory variables (endowment) or the effect of explanatory variables (coefficient), as well as the contribution of specific characteristics to the differences. In this study, we did the decomposition analyses across time and residences.

#### PNC service use across residences

The purpose of the multivariable decomposition analysis was to identify factors that contributed to the differences in PNC service use by residences in both surveys (2016 and 2019). At each survey, rural was coded as "0" and urban was coded as "1".

#### PNC services use over time

The purpose of the multivariable decomposition analysis was to estimate the changes and identify factors that contributed to the changes in PNC service use over the last 3 years or between the EDHS 2016 and 2019 surveys. For this analysis, EDHS 20016 was coded as “0” and EDHS 2019 as “1”.

Multivariate decomposition analysis was done using the “mvdcmp” command to understand the change and determinants of change in PNC service use across time and residence. The Logit-based decomposition analysis technique was used for the analysis of factors contributing to the change in PNC service use and to identify the sources of change across time and residence. To understand the sources of factors contributing to the change in PNC service use between 2016 and 2019, as well as urban and rural in 2016 and 2019, the difference in the composition of explanatory variables (endowment) and the effect of the explanatory variables (coefficient) were considered. Hence, the observed change in PNC uses between 2016 and 2019, as well as in rural and urban areas at each survey, was decomposed into characteristics (E) and coefficients (C). The logit-based difference in PNC service use across residences and over time was decomposed as logit (A)-logit (B) = F (XAβA)- F (XBβB) = $F(\frac{F(XA\beta A)-F(XB\beta A)}{F}+\frac{F((XB\beta A)-(XB\beta B)}{C}$.

Before multivariate decomposition analysis, multi-collinearity between each independent variable was checked. Statistical significance was determined at a p-value of less than 0.05.

### Ethical considerations

The International Review Board of Demographic and Health Surveys (DHS) program data archivists waived informed consent and authorized us to download the data set for this study after the consent paper was submitted to the DHS program. The data set was not shared with or passed on to other organizations, and it was kept confidential. The primary data were collected as per national and international ethical standards. Each woman provided written consent before data collection. Identification, such as a name, was not recorded to maintain confidentiality.

## Results

### Characteristics of participants

In the EDHS 2016, about 49.3% of participants were in rural areas, and 61.9% of participants in urban areas were aged between 24 and 35 years. Two-thirds (65.6%) of rural women had no formal education and 43.4% of urban women had secondary education or more. The majority, 50.7% of rural women and 92.9% of urban women were from poor and rich households, respectively. At the time of the interview, one-fourth of rural households (25.4%) and three-fourths of urban households (34.4%) had radio or television. Below one-third (28.7%) of rural and about two-thirds (67.4%) of urban women had at least four ANC contacts. For the index baby, approximately 31% of rural women and 90.2% of urban women delivered at health facilities.

Similarly, the majority of EDHS 2019 participants were between the ages of 24 and 35 (49.3% of rural and 61.9% of urban). More than half (53.1%) of rural and three-fourths (78.4%) of urban residents were from poor and rich households, respectively. About one-third (29.7%) and 15.8% of rural and urban women, respectively, didn’t receive an ANC during the index pregnancy. Nearly half (47.9%) of rural and above three-fourths (76%) urban women gave birth at health facilities (Table 1).

**Table 1:** Percentage of distribution of socio-demographic and other characteristics of the participants by residences based on the EDHS 2016 and 2019, Ethiopia.

Variables		2016		2019	
		Rural (n = 3,788) (%)	Urban (n = 520) (%)	Rural (n = 1,552) (%)	Urban (n = 553) (%)
Age of women					
	15–24	30.2	22.2	32.2	32.8
	25–34	49.3	61.9	49.1	49.7
	35–49	20.5	15.9	18.7	17.5
Education status					
	No education	65.6	23.0	53.9	25.5
	Primary	30.2	33.6	37.9	45.5
	Secondary+	4.2	43.4	8.2	29
Wealth index					
	Poor	50.7	6.2	53.1	15.4
	Middle	23.4	0.9	23.1	6.2
	Rich	25.9	92.9	23.8	78.4
Marital status					
	Not in union	4.4	7.1	4.6	6.0
	In union	95.6	92.9	95.4	94.0
Religion					
	Christian	31.5	53.7	34.6	39.2
	Muslim	43.7	27.6	38.4	29.2
	Other	24.8	18.7	27.0	31.6
Has radio/television					
	No	74.6	26.5	75.3	34.4
	Yes	25.4	73.5	24.7	65.6
Sex of household head					
	Male	88.2	74.0	89.4	78.8
	Female	11.8	26.0	10.6	21.2
ANC contact					
	No	39.0	7.6	29.7	15.8
	1–3	32.3	25.0	31.9	24.3
	4 or more	28.7	67.4	38.4	59.8
Place of delivery					
	Home	69.0	9.8	52.1	24
	Health Facility	31.0	90.2	47.9	76
Birth order					
	1	18.3	35.6	21.4	30.4
	2–3	28.9	42.6	31.6	41.6
	4 or more	52.8	21.8	47.0	28.0
Sex of children					
	Male	48.5	49.0	51.3	51.9
	Female	51.5	51.0	48.7	48.1
Under five children					
	0	2.2	7.7	3.5	2.1
	1	34.0	55.4	37.3	45.0
	2 or more	63.8	36.9	59.2	52.9

### Change in postnatal care use across residences and over time

**Fig 1:**
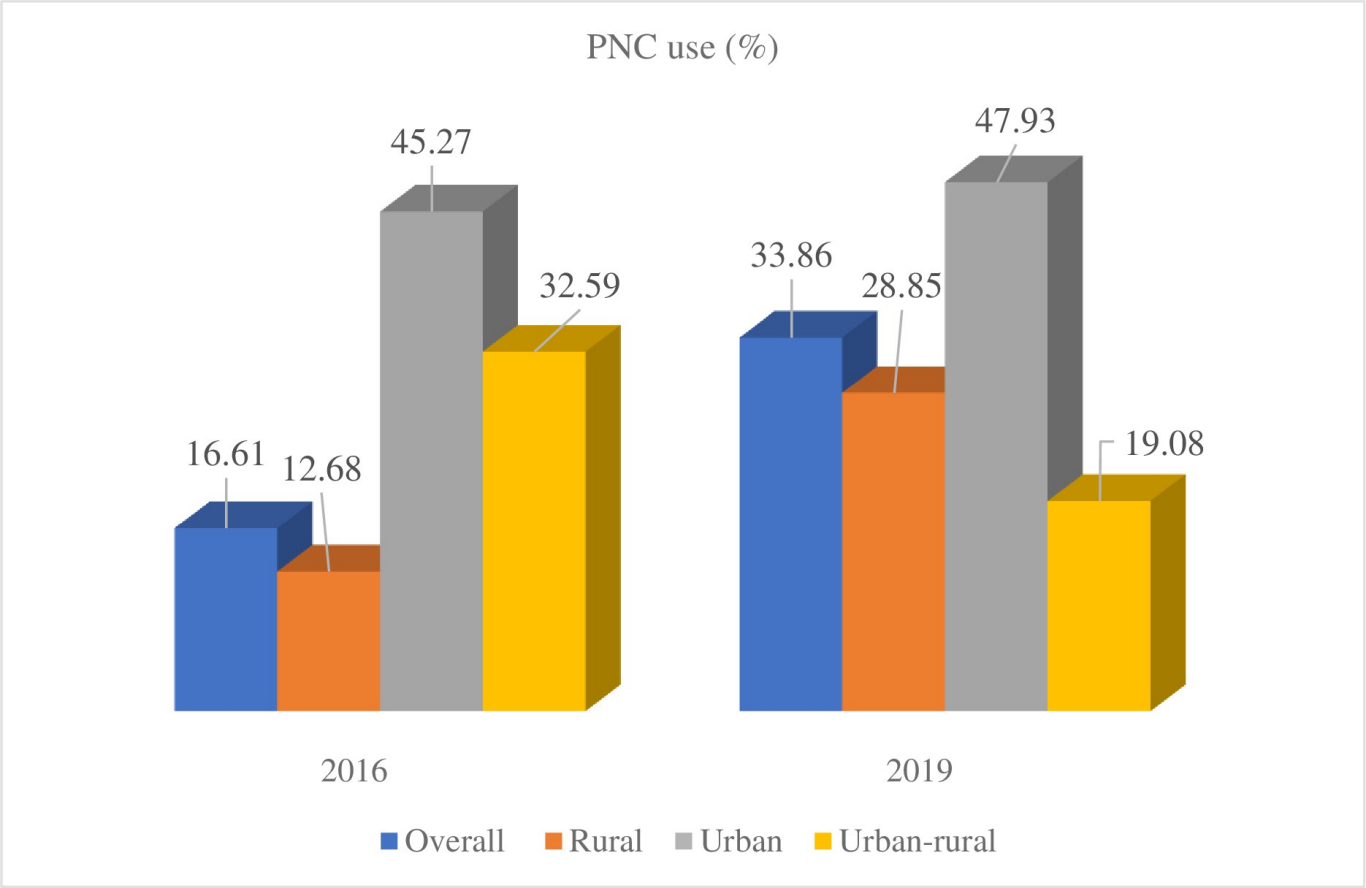
Residencial PNC use among women who gave birth 2 years preceding the survey based on EDHS 2016 and 2019, Ethiopia.

The prevalence of PNC use increased from 16.61% (95% CI [14.48–18.98]) in 2016 to 33.86% (95% CI [28.55–39.62]) in 2019. In both surveys, there was a statistical difference in PNC use between urban and rural women. In EDHS 2016, PNC use increased from 12.68% (95% CI [11.24–14.27]) in rural areas to 45.27% (95% CI [39.54–51.13]) in urban areas. Similarly, in EDHS 2019, PNC uses increased from 28.85% (95% CI [25.13–32.89]) in rural areas to 47.93% (95% CI [37.22–58.84]) in urban areas. However, the overall point difference between urban and rural areas decreased from 32.59% (95% CI [27.40–37.78]) in 2016 to 19.08% (95% CI [13.68–24.47]) in 2019 (Fig 1).

### Residential PNC use by participants’ characteristics

PNC use significantly increased among rural residents from 12.68% (95% CI [11.24–14.27]) in 2016 to 28.85% (95% CI [25.13–32.89]) in 2019, but there was no statistically significant change in PNC use among urban residents between the two surveys. It was 45.27% (95% CI [39.54–51.13]) in 2016 and 47.93% (95% CI [37.22–58.84]) in 2019.

The 2016 EDHS revealed that PNC use increased by 35.6%, 34.2%, 30.9%, 29.1%, 27.4%, 12.3%, and 33.9% in urban areas compared to rural areas for women 25–34 years old, those who had no education, those who were from poor households, those who were married, those who had four or more ANC contacts, those who delivered a baby at a health institution, and those who had two or more under-five children, respectively. Besides, PNC service use increased by 33.2% among urban residents who had no radio or television in the household.

In the EDHS 2019, PNC use increased by 24.7%, 20.7%, and 12.8% among urban residents compared to rural residents when the urban women had two or more under-five children in the household, those who had four or more ANC contacts, and those with primary education, respectively. Urban women from the middle wealth index, those who had 1–3 ANC contacts, and those who had radio or television decreased by 15.4%, 6.7%, and 2.2%, respectively, when compared to rural women.

The chi-square test result showed that in the EDHS 2016, PNC use was associated with educational status, household wealth status, religion, ANC contact, place of delivery, and birth interval among rural areas, and with marital status, ANC, place of delivery, and birth interval among urban areas, p<0.05. In the EDHS 2019, PNC use was associated with educational status, household wealth status, religion, ANC, birth order, and the number of under-five children in rural areas, and with educational status, household wealth status, religion, ANC contacts, and place of delivery in urban areas, p<0.05 (Table 2).

**Table 2:** Residencial PNC service uses distribution by their characteristics among women who gave birth 2 years preceding the survey based on EDHS 2016 and 2019, Ethiopia.

	Variables	PNC use in 2016			PNC use in 2019		
		Rural (n = 3,788) (%)	Urban (n = 520) (%)	Point difference (urban-rural)	Rural (1,552) (%)	Urban (n = 553) (%)	Point difference (urban-rural)
Age of women							
	15–24	13.4	36.7	23.3	34.3	42	7.7
	25–34	12.9	48.5	35.6	26.1	51.8	25.7
	35–49	11.0	44.9	33.9	26.6	48.1	21.5
Education status		***			***	**	
	No education	9.1	40.0	30.9	20.4	30.1	9.7
	Primary	17.6	43.1	25.5	33.1	45.9	12.8
	Secondary+	32.7	49.8	17.1	65	66.8	1.8
Wealth index		***			***	*	
	Poor	8.7	37.8	29.1	16.7	20.5	3.8
	Middle	14.3	21.0	6.7	29.1	13.7	-15.4
	Rich	19.0	46.0	27.0	55.8	56	0.2
Marital status			*				
	Not in union	17.8	28.2	10.4	29.6	28.5	-1.1
	In union	12.4	46.6	34.2	28.8	49.2	20.4
Religion		***			*	**	
	Christian	19.6	49.0	29.4	39.1	68.3	29.2
	Muslim	9.1	37.8	28.7	21.8	50.3	28.5
	Other	10.4	45.6	35.2	25.8	20.6	-5.2
Has radio/television					***	***	
	No	11.6	44.8	33.2	23.2	23.3	0.1
	Yes	16.0	45.5	29.5	46.2	60.8	14.6
Sex of household head							
	Male	12.9	42.5	29.6	28.7	48.2	19.5
	Female	10.9	53.2	42.3	29.8	46.9	17.1
ANC contact		***	***		***	***	
	No	2.8	23.0	20.2	3.7	9.1	5.4
	1–3	13.8	33.3	19.5	30.0	23.3	-6.7
	4 or more	24.8	52.2	27.4	47.5	68.2	20.7
Place of delivery		***	***		***	***	
	Home	1.4	2.8	1.4	2.4	0.2	-2.2
	Health Facility	37.7	50.0	12.3	57.7	63	5.3
Birth order					***		
	1	16.4	37.3	20.9	40.2	51.4	11.2
	2–3	13.2	52.6	39.4	32.3	50.7	18.4
	4 or more	11.1	44.0	32.9	21.4	40.0	18.6
Sex of children							
	Male	12.8	45.1	32.3	28.4	47.2	18.8
	Female	12.5	45.4	32.9	29.3	48.7	19.4
Birth interval		**	*				
	<24 months	6.4	29.0	22.6	14.6	32.8	18.2
	>24 months	12.9	52.5	39.6	28.4	48.6	20.2
	No previous birth	16.5	37.9	21.4	40.2	53.2	13.0
Under five children					***		
	0	23.0	48.2	25.2	32.3	51.3	19.0
	1	15.1	45.1	30.0	39.1	49.8	10.7
	2 or more	11.0	44.9	33.9	22.2	46.2	24.0

Key

* = <0.05

** = < 0.01, and

*** = <0.001.

### Factors that contributed to change in PNC over time

There was a statistically significant change in PNC use between the two surveys: 17.25% (95% CI [14.61–19.90]) increased in 2019 compared to 2016, p<0.001. The decomposition analysis revealed that the majority of the explained change, 60.17% (β = 0.1038 95%CI [0.0936–0.1140]), was due to a change in the composition of explanatory variables between the two surveys. The change in composition of urban residents (β = -0.0100; 95%CI [-0.0198, -0.0002]), receiving 1 to 3 ANC contacts (β = -0.0019; 95%CI [-0.0033, -0.0006]), and religion followers other than Christian or Muslim (β = -0.0048; 95%CI [-0.0076, -0.0020]) reduced the change in PNC use between the two surveys. Whereas, difference in the composition of rich households (β = 0.0040; 95%CI [0.0009–0.0070]), Muslim religion followers (β = 0.0059; 95%CI [0.0027–0.0091]), having radio or television in the household (β = 0.00257;95%CI [0.00001–0.00513]), having four or more ANC contacts (β = 0.0226; 95%CI [0.0137–0.0315]) and health facility delivery (β = 0.0828; 95%CI [0.0721, 0.0935]) increased the change in PNC service use.

The change in PNC use between the two surveys would have increased by 6% if the composition of urban residents stayed the same between the 2016 and 2019 EDHSs. Similarly, if the composition of women who received 1 to 3 ANC visits and those who followed other than Christian or Muslim religions were identical between 2016 and 2019, the change in PNC use would have increased by 1% and 3%, respectively. On the contrary, the change in the prevalence of PNC use between the two surveys would have decreased by 13%, 48%, 3%, 2%, and 2% if the composition of having four or more ANC contacts, health facility deliveries, Muslim religion followers, a rich household, and having radio or television in the household were the same between the 2016 and 2019 EDHSs, respectively.

The remaining 40.34% of the overall change in PNC use between the two surveys was due to changes in the coefficients or behavior of the population towards PNC. The change in PNC service use between the two surveys would have decreased by 40% if the population behavior towards PNC was similar in the two surveys (2016 and 2019 EDHS) (Table 3).

**Table 3:** Change in PNC uptake over time among women who gave birth two years preceding the survey based on EDHS 2016 (n = 4,308) and 2019 (n = 2,105), Ethiopia.

Variables		Difference due characteristics (E)	%	Difference due to coefficient (C)	%
Age of women					
	15–24	1		1	
	25–34	0.0003 (-0.0008, 0.0013)	0.14	-0.0146 (-0.0705, 0.0416)	-8.37
	35–49	-0.0001 (-0.0017, 0.0014)	-0.08	0.0082 (-0.0239, 0.0405)	4.80
Residence					
	Rural	1			
	Urban	-0.0100 (-0.0198, -0.0002) *	-5.79	-0.0156(-0.0352, 0.0041)	-9.01
Education status					
	No education	1		1	
	Primary	0.0019 (0.0038, 0.0077,	1.11	-0.0101 (-0.0411, 0.0209)	-5.85
	Secondary^+^	0.0012 (0.0029, 0.0053)	0.68	-0.0047 (-0.0175, 0.0081)	-2.72
Wealth index					
	Poor	1		1	
	Middle	-0.0002 (-0.0016, 0.0013)	-0.11	-0.0030 (-0.0275, 0.0214)	-1.76
	Rich	0.0040 (0.0009, 0.0070) **	2.31	0.0373 (-0.0140, 0.0891)	21.81
Marital status					
	Not in union	1		1	
	In union	-0.0001(-0.0002, 0.0001)	-0.03	0.0929 (-0.0812, 0.2671)	-53.85
Religion					
	Orthodox	1		1	
	Muslim	0.0059 (0.0027, 0.0091) ***	3.42	-0.0251 (-0.0729, 0.0227)	-14.56
	Other	-0.0048 (-0.0076, -0.0020) **	-2.76	-0.0232 (-0.0568, 0.0105)	-13.42
Has radio/television					
	No	1		1	
	Yes	0.00257 (0.00001, 0.00513) *	1.49	0.0228 (-0.0163, 0.0619)	13.23
Sex of household head					
	Male	1		1	
	Female	-0.0001 (0.0002, 0.0001)	-0.03	0.0007 (-0.0150, 0.0164)	0.42
ANC contacts					
	No	1		1	
	1–3	-0.0019 (-0.0033, -0.0006) **	-1.13	0.0159 (-0.0335, 0.0652)	9.19
	4 or more	0.0226 (0.0137, 0.0315) ***	13.09	0.0289 (-0.0285, 0.0863)	16.77
Place of delivery					
	Home	1			
	Health Facility	0.0828 (0.0721, 0.0935) ***	47.98	0.0083 (-0.0516, 0.0682)	4.80
Birth order					
	1	1			
	2–3	0.0021 (-0.0009, 0.0051)	1.20	-0.0110 (-0.0510, 0.0290)	-6.39
	4 or more	-0.0026 (-0.0097, 0.0046)	-1.48	0.0582 (-0.1538, 0.0374)	-33.73
Sex of children					
	Male	1		1	
	Female	0.0001 (-0.0014, 0.0016)	0.04	0.0110 (-0.0306, 0.0526)	6.38
Under five children					
	0	1		1	
	1	-0.0002 (-0.0045, 0.0041)	-0.12	0.0225 (-0572, 0.1223)	18.85
	2 or more	0.0004 ((-0.0043, 0.0051)	0.23	0.0448 (-0.1031, 0.1927)	25.95
Total		0.1038 (0.0936,0.1140) ***	60.17	0.0687 (0.0474, 0.0900) ***	39.83

Key: 1 reference

* significant at <0.05

** significant at <0.01, and

*** significant at <0.001.

### Factors contributed to the change in PNC across the residence

In the EDHS 2016, there was a 32.59% (95% CI [27.40–0.3778]) increase among urban areas compared to their counterpart rural areas at P<0.001. The decomposition analysis revealed that differences in the composition of explanatory variables accounted for 88.99% (β = 0.2901; 95%CI [0.2273–0.3528]) of the disparity across residences in 2016. The remaining overall change, 11.01%, was attributed to differences in the coefficient of explanatory variables across the residences but was not statistically significant (β = 0.0359; 95%CI [-0.0173–0.0891]). In the EDHS 2019, there was a 19.08% (95% CI [13.68–24.47]) increase among urban areas compared to rural areas at P<0.001. The decomposition analysis showed that 110.07% (β = 0.0210 95%CI [0.1561–0.2638] of the explained change across the residence was attributable to differences in the composition of explanatory variables. About 11% of the contracting effect in overall change was attributed to differences in explanatory variable coefficients across residences, but this was not statistically significant (β = -0.0192; 95%CI [-0.0791–0.0407]) (Table 4).

**Table 4:** Summary of the result from urban-rural decomposition analysis based on EDHS 2016 (rural = 3,788 and urban = 520) and 2019(rural = 1,552 and urban = 553), Ethiopia.

	Estimated 95%CI	
	2016	2019
Estimated PNC use in urban	45.27% (39.54–51.13) ***	47.93% (37.22, 58.84) ***
Estimated PNC use in rural	12.68% (11.24–14.27) ***	28.85% (25.13, 32.89) ***
Total difference (R)	32.59% (27.40, 0.3778) ***	19.08% (13.68, 24.47) ***
Difference due characteristics (E)	0.2901 (0.2273–0.3528) ***	0.0210 (0.1561, 0.2638) ***
Difference due to coefficient (C)	0.0359 (-0.0173, 0.0891)	0.0192 (-0.0791, 0.0407)

Key: *** P <0.001

Only the differences in the composition of explanatory variables between the two surveys were statistically significant for the disparity in PNC use by residences. If the explanatory variable’s composition had been equalized between urban and rural people, the explained urban-rural disparity in PNC use would have been reduced by 89 and 110 percent, respectively, in 2016 and 2019. The change due to coefficients is not statistically significant in both surveys; thus, the detailed result of each variable’s decomposition is not included in the table.

In 2016, the difference in the composition of female household heads, health facility delivery, and two to three and four birth orders significantly contributed to the urban-rural disparity in PNC use. If the composition of female household heads and institutional delivery in urban and rural areas were identical, the difference in PNC service use would have decreased by 5% (β = 0.0147; 95%CI [0.0048–0.0246]) and 83% (β = 0.2720; 95%CI [0.2109–0.3317]), respectively. Similarly, if the composition of women with two to three birth orders had remained identical in urban and rural areas, the difference in the use of PNC service would have decreased by 6% (β = 0.0180; 95%CI [0.0055–0.0306]). On the other hand, the gap in PNC service use between rural and urban residents would have increased by 12% if the composition of women with four or more birth orders was the same between rural and urban (β = -0.0399; 95% [-0.0783, -0.0015]).

In 2019, the difference in the composition of women from households’ middle wealth index (β = -0.0280, 95%CI [-0.0557- -0.0002]) between the two settings was a gap-narrowing variable. Whereas, the difference in the composition of Muslim religious followers (β = 0.0073; 95%CI [0.0009–0.0138], having four or more ANC contacts (β = 0.0358 95%CI [0.0142–0.0574]), and health facility delivery (β = 0.1539; 95%CI [0.1288–0.1790]) were gap-widening variables. The urban-rural gap in PNC use in 2019 would have increased by 15% if the composition of women from households with a middle wealth index were identical between urban and rural areas. Whereas, if the composition of Muslim religious followers, four or more ANC contacts, and health facility delivery were identical in urban and rural areas, the difference in use of PNC service would have decreased by 4%, 18%, and 81%, respectively (Table 5).

**Table 5:** Residential decomposition changes in PNC uptake among women who gave birth 2 years preceding the survey based on EDHS 2016 (rural = 3,788 and urban = 520) and 2019(rural = 1,552 and urban = 553), Ethiopia.

Variables		EDHS 2016		EDHS 2019	
Age of women		Difference due characteristics (E)	%	Difference due characteristics (E)	%
	15–24	1		1	
	25–34	0.0026 (-0.0071, 0.0123)	0.80	0.0003 (-0.0002,0.0008)	0.14
	35–49	0.0020 (-0.0033, 0.0073)	0.60	-0.0003(-0.0016, 0.0009)	-0.18
Education status					
	No education	1		11	
	Primary	-0.0008 (-0.0036, 0.0020)	-0.24	0.0011 (-0.0067, 0.0088)	0.55
	Secondary^+^	0.0020 (-0.0332, 0.0372)	0.62	-0.0051 (-0.0297, 0.0195)	-2.67
Wealth index					
	Poor	1		1	
	Middle	-0.0262 (-0.0821, 0.0298)	-8.03	-0.0280 (-0.0557, -0.0002) *	-14.66
	Rich	0.0193 (-0.0752, 0.1138)	5.92	0.0291 (-0.0247, 0.0829)	15.25
Marital status					
	Not in union	1		1	
	In union	-0.0026 (-0.0058, 0.0006)	-0.80	-0.0006 (-0.0024, 0.0013)	-0.29
Religion					
	Christian	1		1	
	Muslim	0.0077 (-0.0041, 0.0195)	2.36	0.0073 (0.0009, 0.0138) *	3.84
	Other	-0.0026 (-0.0079, 0.0028)	-0.79	-0.0038 (-0.0085, 0.0010)	-1.98
Has radio/television					
	No	1		1	
	Yes	-0.0100(-0.0479, 0.0280)	-3.05	0.0279 (-0.0046, 0.0603)	14.63
Sex of household head					
	Male	1		1	
	Female	0.0147 (0.0048, 0.0246) **	4.51	0.0001 (-0.0096, 0.0094)	-0.03
ANC contact					
	No	1		1	
	1–3	0.0012 (-0.0101, 0.0125)	0.36	-0.0014 (-0.0095, 0.0068)	-0.71
	4 or more	0.0296 (-0.0266, 0.0859)	9.09	0.0358 (0.0142, 0.0574) **	18.29
Place of delivery					
	Home	1		1	
	Health Facility	0.2720 (0.2109, 0.33317) ***	83.45	0.1539 (0.1288, 0.1790) ***	80.66
Birth order					
	1	1		1	
	2–3	0.0180 (0.0055, 0.0306) **	5.53	-0.0078 (-0.0186, 0.0029)	-4.11
	4 or more	-0.0399 (-0.0783, -0.0015) *	-12.24	0.0057 (-0.0241, 0.0355)	2.97
Sex of children					
	Male	1		1	
	Female	0.00003 (-0.00024, 0.00031)	0.01	-0.0005 (-0.0046, 0.0055)	-0.25
Under five children					
	0	1		1	
	1	-0-.0096 (-0.0385, 0.0192)	-2.96	0.0035 (-0.0190, 0.0260)	1.82
	2 or more	0.0125 (-0.0262, 0.0513)	3.85	-0.0075 (-0.0259,0.0109)	-3.95
Total	-	0.2901 (0.2273, 0.3528) ***	88.99	0.02100 (0.1561, 0.2638) ***	110.07

Key: 1 reference, * significant at <0.05

** significant at <0.01, and

*** significant at <0.001.

## Discussion

Over the last two decades, Ethiopia has achieved significant progress in improving women’s and children’s health [4, 5]. However, Ethiopia must reduce maternal death by five times and newborn mortality by more than three times, commencing in 2016, to meet SDG 3 by 2030. The majority of maternal and neonatal deaths occur during the first two days after birth. PNC, particularly in the first two days after birth, is crucial to avert it, but it has remained low in Ethiopia as compared to other maternal health service coverage. Hence, this study aimed to examine PNC use change and to identify factors that contributed to the difference in PNC service uptake across the residences and over time using EDHS 2016 and 2019. The findings of this study revealed that there was a statistically significant change in PNC service use over three years in Ethiopia. PNC use has increased by 17.25% in 2019. This finding was supported by previous studies conducted in Ethiopia [19] and Bangladesh [21]. The authors agreed that the increasing PNC service uptake in Ethiopia recently could be attributable to increased global and national efforts to improve maternal and child health. Following the SDG agenda of lowering maternal mortality and eliminating avoidable newborn mortality, Ethiopia launched the national health sector transformation plan 1 [13] and reproductive health strategy [14] in 2016, intending to reach 95% uptake of maternal health services (ANC, institutional delivery, and PNC) by 2020, which may positively enforce expanding maternal health service accessibility, enhancing monitoring, and motivating the overall health system to achieve the goal.

This study also found that there was a significant urban-rural disparity in PNC use in both surveys. In 2016, PNC use increased from 12.68% in rural areas to 45.27% in urban areas, while in 2019, it increased from 28.85% to 47.93%. This finding was supported by previous studies conducted in Ethiopia [19, 22], Malawi [23], and India [9]. The urban-rural gap in PNC service uptake may be due to inadequate access to health facilities, inadequate infrastructure, lack of media exposure, low women’s autonomy, and low education as well as low economic status [23, 24]. On the other hand, metropolitan populations are relatively wealthy and better educated, and private and governmental health facilities are likewise concentrated, allowing greater access to the modern healthcare system. In Ethiopia, urban-rural disparity in access to healthcare resources and health facilities is deeply ingrained, and urban areas are better positioned to access quality healthcare [25]. Furthermore, a study reported that rural women are uneducated and lack decision-making power regarding the use of maternal health services [26]. In our study, women from rich households more than tripled in urban settings as compared to rural settings. Moreover, three-fourths of urban residents and only one-fourth of rural residents had radio or television in their households.

The urban-rural disparity in PNC use decreased from 32.59% in 2016 to 19.08% in 2019. Moreover, PNC use significantly increased among rural residents from 12.68% in 2016 to 28.85% in 2019, but there was no statistically significant change among urban residents between the two surveys. The decomposition analysis also showed the change in the composition of urban residences had a significant counteracting effect (-6%) on the change in PNC use over time. One possible explanation is that the increased focus on enhancing the quality and accessibility of primary healthcare for women and children, particularly in rural areas, is attributed to various initiatives by governmental and non-governmental organizations. Innovative interventions such as the Pregnancy Women Conference and the expansion of the women’s development army strategy have significantly contributed to the recent improvement in maternal health services in rural settings [27, 28].

The decomposition analysis of this study revealed that the difference between urban and rural areas in both surveys was significantly attributed to only the differences in the composition of explanatory variables (endowment). On the other hand, about 60% and 40% of the percentage change in PNC use in 2019 was due to differences in the composition of explanatory variables and behavioral (coefficient) change of the population towards PNC, respectively. This study revealed that the majority of urban-rural disparity and change over time in PNC use was attributed to the difference in composition of ANC and health facility delivery across residences and over time. Studies in low- and middle-income countries, including Ethiopia, indicated that women with ANC contacts and those who gave birth at health facilities were more likely to utilize PNC services [29–32]. The reason for this could be that increased ANC contacts and health facility deliveries offer enhanced opportunities for counseling and education on postnatal danger signs. Such crucial information, possibly missed in earlier visits and for birth at home, is essential for raising awareness about the benefits of PNC services. Therefore, the cumulative effect of comprehensive ANC and health facility deliveries contributes to a more informed understanding of postnatal health, potentially explaining the widening urban-rural gap in PNC service use [33].

This study revealed that about 5% of the percentage change in PNC use in urban residences was due to the difference in the composition of female household heads in 2016. A study in Ethiopia showed that women from female-headed households are more likely to use PNC services [34]. In another study conducted in four African countries, including Ethiopia, women who were female heads of household were more likely to receive maternal health services [35]. Likewise, a study in India found that women’s freedom of movement contributed to inequality in the use of PNC [36]. The reason for this could be that being the head of the household is a proxy for the woman’s autonomous decision-making status in the household.

This study revealed that the difference in composition of first- to second-birth orders accounted for approximately 6% of the percentage change in PNC use in urban residences, while the difference in composition of four or more birth orders had a contracting effect (-12%) in 2019. The finding was supported by the previous study [36]. A study conducted in rural Ethiopia also showed that the use of maternal health services decreased with increasing birth orders [34]. Studies in Africa revealed that high birth order negatively affects maternal health-seeking behavior and service use [37, 38]. A study conducted in rural India revealed that the likelihood of PNC service use decreases as birth order increases [39]. A possible justification might be that higher birth order is a symbol of bigger families. Evidence showed that high birth orders and large family sizes are characteristics of poor families, which may worsen access to healthcare [40]. Another evidence revealed that having more children may result in resource constraints, which may have a detrimental impact on healthcare use [41].

The differences in the composition of religion played an important role in promoting disparity in PNC use across residences in 2019 and over time. Previous studies in Ethiopia showed that PNC use varied by religion [34, 42, 43]. The urban-rural disparity in PNC service use by religion was also documented in the previous study conducted in India [36]. One potential explanation for this variation could be attributed to religious differences in the practice of indoor confinement for women following childbirth [43]. Women’s autonomy and cultural beliefs may change over time as a result of education, urbanization, and media exposure, all of which are linked to maternal health service use [44].

In this study, the difference in the wealth index composition significantly influenced the disparity in PNC use across residences and over time. In 2019, the difference in composition of the Middle wealth index had a counteracting effect (-15%) on the percentage change in PNC use in urban residences. On the other hand, the change in the composition of the rich wealth index between the two surveys accounted for over 2% of the percentage change in PNC use in 2019. This finding was supported by a previous study [36]. Even if maternal healthcare is free in Ethiopia, rural women may live far from the health facility and must travel long distances to get there; however, being wealthy may allow them to pay for transportation and other fees as well as access to mass media [45].

This study revealed that nearly 2% of the percentage change in PNC use in 2019 was due to the change in the composition of households having radio and/or television. In Ethiopia, radio and television are two key mass media for distributing maternal health and other information. Access to information via various media empowers people to make their own decisions, make healthy lifestyle choices, and raise knowledge of obstetric-related risks and consequences [46], which enhances PNC service use [47].

In this study, about 40% of the percentage change in PNC use in 2019 was attributed to behavioral change in the population towards PNC between the two surveys, but there was no specific variable associated with it. This implies that behavioral changes among all participants, rather than just a subset, have a positive impact on PNC use change.

The data for this study come from large, nationally representative surveys. This study provided evidence of a change in PNC service use across residences and over time, as well as identified the factors contributing to the change. This may help policymakers to ensure equitable PNC service provision to women in need in Ethiopia by focusing on the above key factors. Nonetheless, some important variables are missing from this analysis because they were not collected at the primary source. Furthermore, since EDHS are cross-sectional surveys and rely on self-reported data, recall bias may be possible. Hence, further studies are needed that incorporate an alternative methodology to the decomposition analysis.

## Conclusion

The findings of this study showed that significant urban-rural disparity was observed in PNC service use in both surveys; the direction of the disparity in the 2016 and 2019 surveys was similar; urban women were more likely to use PNC service. This study revealed that the difference in the composition of explanatory variables was significantly attributed to the urban-rural disparity in both surveys. Particularly, ANC contact and place of delivery at both surveys, sex of the household head, and birth order in 2016, whereas wealth status and religion in 2019, significantly contributed to the urban-rural disparity in PNC use. The study also revealed that there was a statistically significant change in PNC service use between 2016 and 2019, with a high prevalence in 2019. The majority of the change (60%) over time was due to differences in the composition of explanatory variables. Particularly, residence, wealth status, religion, radio or television, ANC contacts, and place of delivery were statistically significant contributors to the observed change. The remaining (40%) overall change was due to the difference in unknown behaviors (coefficient) of the population towards PNC. Therefore, strengthening public interventions aimed at improving PNC use in rural areas is essential. Particularly, four or more ANC contacts and delivery at a health facility played a major role in explaining the gap in PNC services across the residences and over time in Ethiopia, highlighting the need to expand interventions that promote ANC and health facility delivery services among rural residents. Moreover, implementing initiatives that enhance the household economy and empower women as household heads may help to narrow the gap in PNC service use across residences as well as increase its uptake in Ethiopia. Furthermore, expanded media accessibility and public behavior-change interventions towards PNC are important to sustain the improvement of PNC use in Ethiopia.
